# Impulsive choice in two different rat models of ADHD—Spontaneously hypertensive and *Lphn3* knockout rats

**DOI:** 10.3389/fnins.2023.1094218

**Published:** 2023-01-26

**Authors:** Monica S. Carbajal, Asiah J. C. Bounmy, Olivia B. Harrison, Hunter G. Nolen, Samantha L. Regan, Michael T. Williams, Charles V. Vorhees, Helen J. K. Sable

**Affiliations:** ^1^Department of Psychology, University of Memphis, Memphis, TN, United States; ^2^Department of Pediatrics, University of Cincinnati College of Medicine, Cincinnati, OH, United States; ^3^Division of Neurology, Cincinnati Children’s Hospital Medical Center, Cincinnati, OH, United States

**Keywords:** externalizing behavior, response inhibition, delay-discounting, spontaneously hypertensive rat (SHR), *Adgrl3*, *Lphn3* KO rat, latrophilin 3, attention-deficit/hyperactivity disorder (ADHD)

## Abstract

**Introduction:**

Impulsivity is a symptom of attention-deficit/hyperactivity disorder (ADHD) and variants in the *Lphn3 (Adgrl3*) gene (OMIM 616417) have been linked to ADHD. This project utilized a delay-discounting (DD) task to examine the impact of *Lphn3* deletion in rats on impulsive choice. “Positive control” measures were also collected in spontaneously hypertensive rats (SHRs), another animal model of ADHD.

**Methods:**

For Experiment I, rats were given the option to press one lever for a delayed reward of 3 food pellets or the other lever for an immediate reward of 1 pellet. Impulsive choice was measured as the tendency to discount the larger, delayed reward. We hypothesized that impulsive choice would be greater in the SHR and *Lphn3* knockout (KO) rats relative to their control strains - Wistar-Kyoto (WKY) and *Lphn3* wildtype (WT) rats, respectively.

**Results:**

The results did not completely support the hypothesis, as only the SHRs (but not the *Lphn3* KO rats) demonstrated a decrease in the percent choice for the larger reward. Because subsequent trials did not begin until the end of the delay period regardless of which lever was selected, rats were required to wait for the next trial to start even if they picked the immediate lever. Experiment II examined whether the rate of reinforcement influenced impulsive choice by using a DD task that incorporated a 1 s inter-trial interval (ITI) immediately after delivery of either the immediate (1 pellet) or delayed (3 pellet) reinforcer. The results of Experiment II found no difference in the percent choice for the larger reward between *Lphn3* KO and WT rats, demonstrating reinforcement rate did not influence impulsive choice in *Lphn3* KO rats.

**Discussion:**

Overall, there were impulsivity differences among the ADHD models, as SHRs exhibited deficits in impulsive choice, while the *Lphn3* KO rats did not.

## Introduction

Attention-deficit/hyperactivity disorder (ADHD) is a highly prevalent neurodevelopmental disorder characterized by impulsivity, inattention, and hyperactivity (American Psychological Association [APA], 2013). ADHD is a commonly diagnosed in childhood but can continue into adulthood (Weibel et al., 2020) and is often comorbid with other externalizing disorders (Faraone et al., 2003; Palacio et al., 2004; Frick and Nigg, 2012; Hansen et al., 2018). Based on the 2016 National Parent Survey, about 9.8% of children are diagnosed with ADHD and 6 in 10 children with ADHD had at least one other mental, emotional, or behavioral disorder (Bitsko et al., 2022).

Although many factors in the environment can contribute to the development of ADHD, the literature has shown that genetics can help explain ADHD variability (Spencer et al., 2007). Recent data has shown a linkage of ADHD and other externalizing behaviors with markers on chromosome 4q13.2 (Arcos-Burgos et al., 2004; Acosta et al., 2008; Arcos-Burgos and Muenke, 2010). Mapping of this region has revealed that variants in the *Lphn3* (*Adgrl3*) gene (OMIM 616417) predispose individuals to ADHD (Acosta et al., 2008, 2011, 2016) and predict ADHD severity and response to treatment (Arcos-Burgos et al., 2010; Acosta et al., 2011; Bruxel et al., 2015). Similar studies in other populations have also found that *Lphn3* gene variants contribute to ADHD susceptibility (Ribases et al., 2011; Hwang et al., 2015; Gomez-Sanchez et al., 2016; Martinez et al., 2016; Huang et al., 2019; Kappel et al., 2019; Puentes-Rozo et al., 2019).

Research in animal models has also provided some corroborating evidence for the role of *Lphn3* in ADHD. For example, zebrafish that lack *lphn3.1* (one of two *Lphn3* orthologs) were found to be hyperactive (Lange et al., 2012, 2018)–an effect that was attenuated by the ADHD medications methylphenidate and atomoxetine (Lange et al., 2012). The down-regulation of *lphn3.1* in zebrafish caused a misplacement of dopamine (DA) (but not norepinephrine or serotonin) neurons (Lange et al., 2012) and decreased locomotor sensitivity to DA agonists and antagonists (Lange et al., 2018). Likewise, *Lphn3*^–/–^ knockout (KO) mice and rats are also hyperactive (Wallis et al., 2012; Regan et al., 2019), and they have been shown to be impaired on a facet of impulsivity called impulsive action (Mortimer et al., 2019; Sable et al., 2021) which is the inability to inhibit a prepotent motor response (Bari and Robbins, 2013; MacKillop et al., 2016). The exact mechanism whereby alterations in *Lphn3* gene expression alters catecholamine neurotransmission is still being investigated. However, in both *Lphn3* mutant mice and rats, the expression levels of the DA transporter (DAT) gene (*Slc6a3*) and the protein itself differ from wildtype controls. Adult *Lphn3* KO rats have increased DAT expression (Regan et al., 2019) and increased reuptake of DA (i.e., functional implication for increase in DAT) (Regan et al., 2020) in the dorsal striatum. Likewise, adult *Lphn3*^–^*^/^*^–^ mice demonstrate overexpression of *Slc6a3* in whole brain (Wallis et al., 2012) and in the dorsal striatum (Pramod et al., 2013), but downregulation of *Slc6a3* in the prefrontal cortex (PFC) (Orsini et al., 2016; Mortimer et al., 2019). DAT expression is associated with ADHD (see Pramod et al., 2013 for review), including the site of action for many ADHD medications (Fone and Nutt, 2005; Gerlach et al., 2013; Faraone, 2018), so these results are particularly noteworthy.

While previous research has demonstrated *Lphn3* contributes to hyperactivity and deficits in impulsive action, this project examined the impact of *Lphn3* deletion in rats on a different facet of impulsivity. Using a delay-discounting (DD) task, we assessed impulsive choice, which is the inability to delay gratification (Reynolds et al., 2002). We also report “positive control” measures for the same behavioral assay in spontaneously hypertensive rats (SHRs) which have also been proposed as an animal model of ADHD (Prediger et al., 2005; Sagvolden et al., 2005b,^2009^; Kantak et al., 2008; Meneses et al., 2011; Sagvolden and Johansen, 2012; Garcia and Kirkpatrick, 2013; Natsheh and Shiflett, 2018) and have previously exhibited DD deficits (Fox et al., 2008; Aparicio et al., 2019; Sjoberg et al., 2021). Compared to their control strain, Wistar-Kyoto (WKY) rats, SHRs also exhibit hyperactivity and inattention (Russell, 2011; Sagvolden and Johansen, 2012) as well as deficits in impulsive action (Sable et al., 2021; González-Barriga and Orduña, 2022). Here, we expected to observe more impulsive choice in the SHR and *Lphn3* KO rats represented by their tendency to choose the small, immediate reward more often that the larger, delayed reward relative to their control strains, WKY and *Lphn3* WT rats, respectively.

## Experiment I method

### Subjects

Subjects consisted of 24 SHR (12 male, 12 female) and 24 WKY rats (12 male, 12 female) along with 32 *Lphn3* KO rats (16 male, 16 female) and 33 *Lphn3* WT rats (16 male, 17 female). The SHRs and WKYs were shipped in a single cohort from Charles River (Kingston, NY, USA) at 45 ± 2 days old. The *Lphn3*^–^*^/^*^–^ rats were generated at the Cincinnati children’s transgenic animal and genome editing core by using CRISPR/Cas9 technology (Regan et al., 2019). Once genotypes were confirmed, the KO rats were shipped in three cohorts to the University of Memphis, along with their WT controls, at 40 ± 10 days old.

All rats were housed in same-sex groups of 2–3 per cage in standard plastic cages with corn cob bedding and *ad libitum* tap water in a room with a 12 h reverse light/dark cycle (lights off 7:00 am). Rats were on free feed (Teklad, 2018) until 60 days old, after which they were put on a food restriction schedule to maintain 85–90% of their free-feeding weight so that they would respond for food rewards during behavioral testing. Body weights at the start of operant testing are presented in Supplementary Table 1.

### Apparatus

Behavioral testing was performed in 10 automated, rat operant chambers (Med Associates Inc., St. Albans, VT, USA) housed in sound attenuating wooden boxes equipped with a fan for ventilation. The test chambers measured 17.5 cm tall with a 24 cm × 20 cm stainless steel grid floor resting above a tray filled with corn cob bedding. Dustless grain-based precision pellets (45 mg; Bio-Serv, Flemington, NJ, USA) were dispensed into a food magazine centered 2.5 cm above the floor. A retractable response lever with a cue light above was located on both sides of the food magazine and a house light was located on the opposite wall. White noise was presented during testing to minimize disruption from outside sounds. Med-PC V software (Med Associates) was used to conduct the testing programs and record data.

### Procedure

#### Autoshaping and fixed ratio training

All rats were first trained to lever press for food using an autoshaping program, which was followed by a fixed ratio training program. The former was used to establish the lever press response, and the latter alternated the response requirement every five trials to ensure that no rat exhibited a side preference for either lever. Additional details about the autoshaping and fixed ratio training programs have been previously published (Sable et al., 2021).

#### Delay-discounting

During each DD session, the rat was given the choice to press one lever for one pellet delivered immediately or the other lever for three pellets delivered after 0, 4, 8, 12, or 16 s. As such, one lever was always the immediate lever (0 s) and the delay on the other lever progressively increased every 10 trials using the order of delays presented above for a total of 50 trials/session. Trial lengths were such that if the rat pressed the lever leading to the smaller, but immediate, reward, the next trial did not begin until the delay period on the other lever had elapsed. This ensured that the overall session length was the same for all rats. Rats completed 25 sessions.

### Design and analyses

The data from the SHR/WKY rats were analyzed separately from KO/WT data.

#### Percent choice larger reward

The percent choice for the larger, delayed reward for the 25 sessions was averaged across blocks of 5 days to yield five, 5-day testing blocks. To simplify the omnibus analyses, only data from the first testing block (i.e., acquisition phase) and the last testing block (i.e., maintenance phase) were included. The independent variables included in the omnibus analysis were strain (SHR vs. WKY) or genotype (KO vs. WT), sex (male vs. female), delay (0, 4, 8, 12, and 16 s), and phase (acquisition vs. maintenance). Thus, each analysis was a mixed 2 (strain or genotype) × 2 (sex) × 5 (delay) × 2 (phase) mixed ANOVA where strain/genotype and sex were between-subjects factors and delay and phase were repeated-measures factors.

#### Slope/area under curve

The slope of the discounting curve and area under the curve (AOC) during the acquisition and maintenance phases were analyzed separately using a mixed 2 (strain or genotype) × 2 (sex) × 2 (phase) ANOVA where strain/genotype and sex were between-subjects factors and phase was a repeated-measures factor. The slope was determined by calculating rise/run based on the shortest (0 s) and longest delay (16 s) of the discounting curve for each rat. The AOC was the composite area of the parametric space beneath the percent choice for the larger reward at each delay. Unlike slope, AOC uses all delays and therefore accounts for fluctuations choice behavior more effectively than slope (Myerson et al., 2001).

## Experiment I results

If a rat did not demonstrate 60% choice for the larger reward at the 0 s delay during the final maintenance phase, it was determined that the rat had not learned to differentiate between the levers associated with the small versus large reward (i.e., the rat had not learned the task). Thus, these data were not included in the final analyses. Specifically, data from 4 SHRs (1 male, 3 female), 3 WKY rats (3 male, 0 female), 3 *Lphn3* KO rats (2 male, 1 female), and 6 *Lphn3* WT rats (4 male, 2 female) were not included leaving final *n*’s of 20 SHRs (11 male, 9 female), 21 WKYs (9 male, 12 female), 29 *Lphn3* KOs (14 male, 15 female), and 27 *Lphn3* WTs (12 male, 15 female).

If a sphericity violation was found for any within-subjects effect, a Greenhouse–Geisser correction was used to reduce the risk of a Type I error because ε < 0.75 in all cases (Maxwell and Delaney, 1999). In the interest of brevity, only significant genotype- and strain-related main effects and interactions are reported. There were no significant strain × sex or genotype × sex interactions for any of the dependent variables, so the results are presented collapsed across sex.

### SHR/WKY

**FIGURE 1:**
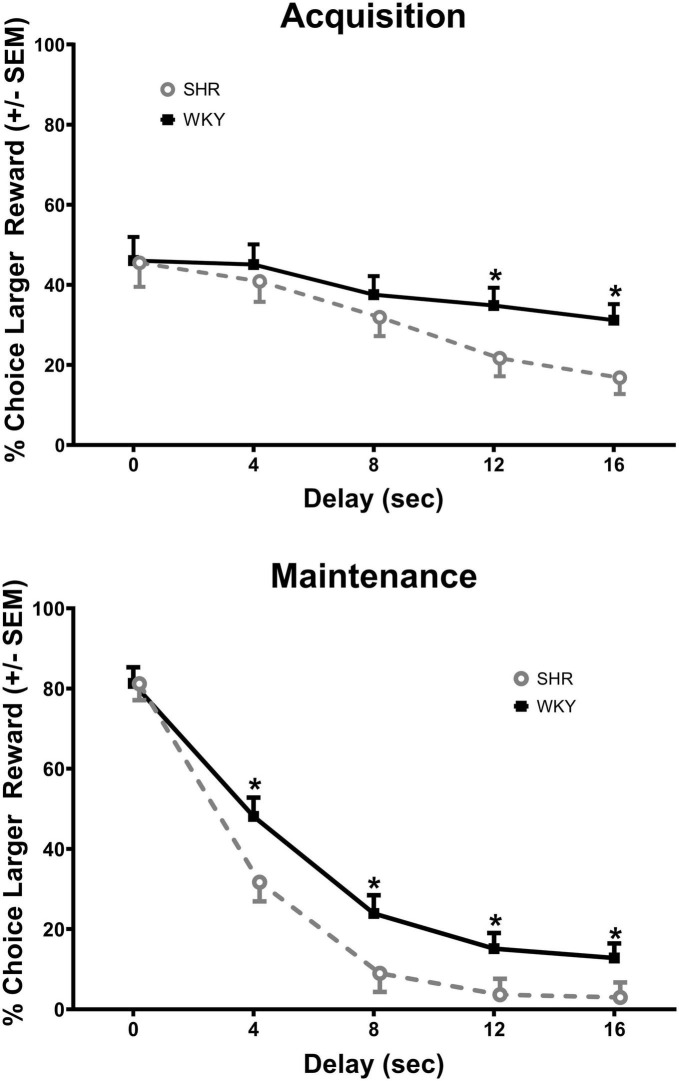
Percent choice for the larger reward during acquisition **(top)** and maintenance responding **(bottom)** for the spontaneously hypertensive rat (SHR) and WKY rats with equal trial lengths. The SHRs discounted the larger reward significantly more than the WKY rats at the longest two delays of acquisition and at all delays during maintenance, *p < 0.05.

**FIGURE 2:**
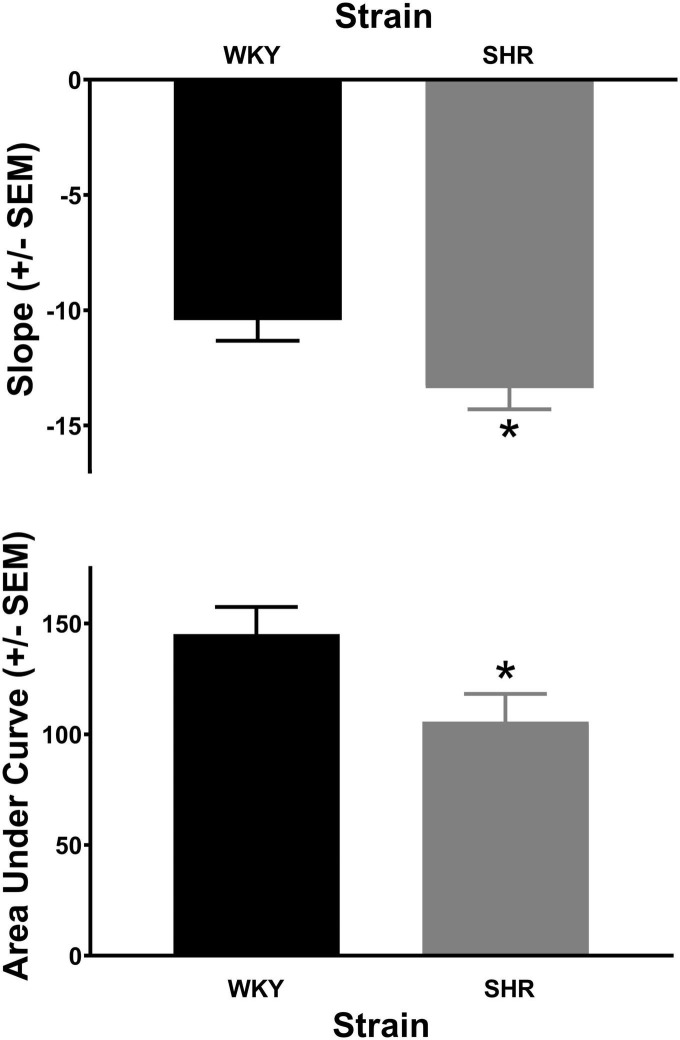
The slope of the discounting curve **(top)** was significantly more negative for the spontaneously hypertensive rat (SHR) versus WKY rats, while the area under the discounting curve **(bottom)** was significantly less for the SHR versus WKY rats, *p < 0.05. The trial lengths were the same regardless of which lever was selected.

The analysis of the percent choice for the larger reward revealed a significant main effect of strain [*F*(1, 37) = 4.269, *p* = 0.046] and significant interactions of strain × delay [*F*(1.671, 61.840) = 3.813, *p* = 0.034] and strain × delay × phase [*F*(2.459, 90.975) = 2.984, *p* = 0.045]. As can be seen in Figure 1, during acquisition (top panel) the SHRs discounted the larger reward significantly more than the WKY rats during the 12 (*p* = 0.031) and 16 (*p* = 0.012) s delays. During maintenance, this effect was present during the 4 (*p* = 0.014), 8 (*p* = 0.018), 12 (*p* = 0.030), and 16 (*p* = 0.044) s delays. The greater discounting by the SHRs was also evident in analyses of slope and AOC, where a main effect of strain was found in both cases [*F*(1, 37) = 5.250, *p* = 0.028 and *F*(1, 37) = 5.016, *p* = 0.031, respectively]. As seen in Figure 2, the slope of the discounting curve was significantly more negative, and the AOC was significantly smaller for the SHRs versus the WKY rats.

### *Lphn3* KO/WT

**FIGURE 3:**
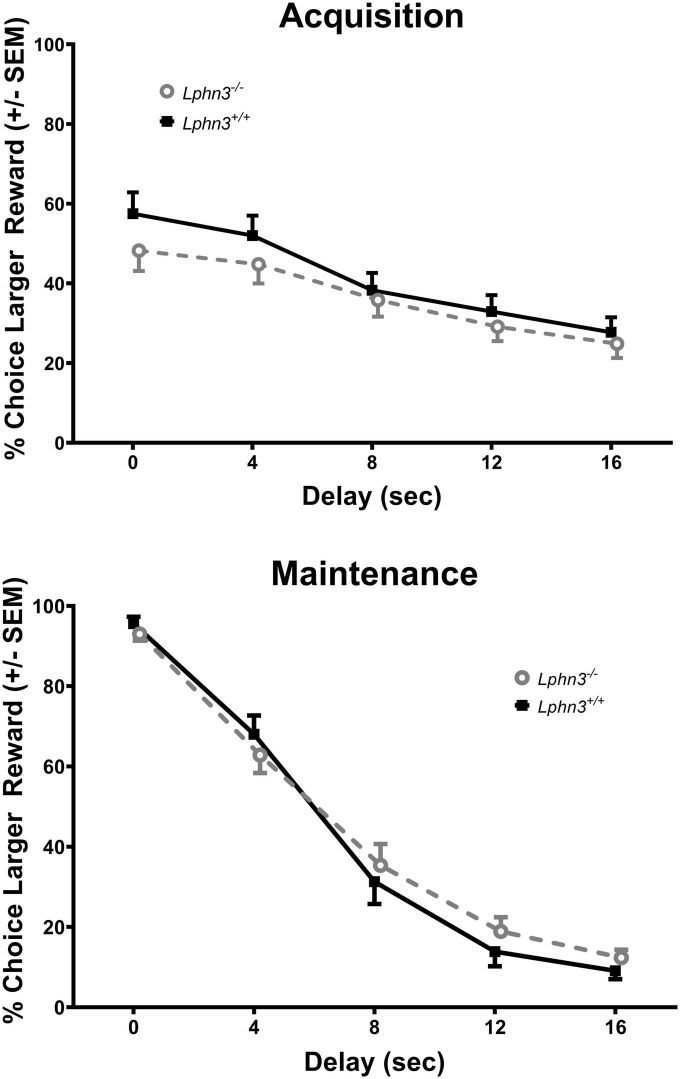
Percent choice for the larger reward during acquisition **(top)** and maintenance responding **(bottom)** for the *Lphn3*^–^*^/^*^–^ (knockout) and *Lphn3*^+^*^/^*^+^ (wildtype) rats with equal trial lengths. Genotype had no effect of on delay-discounting (DD) performance.

**FIGURE 4:**
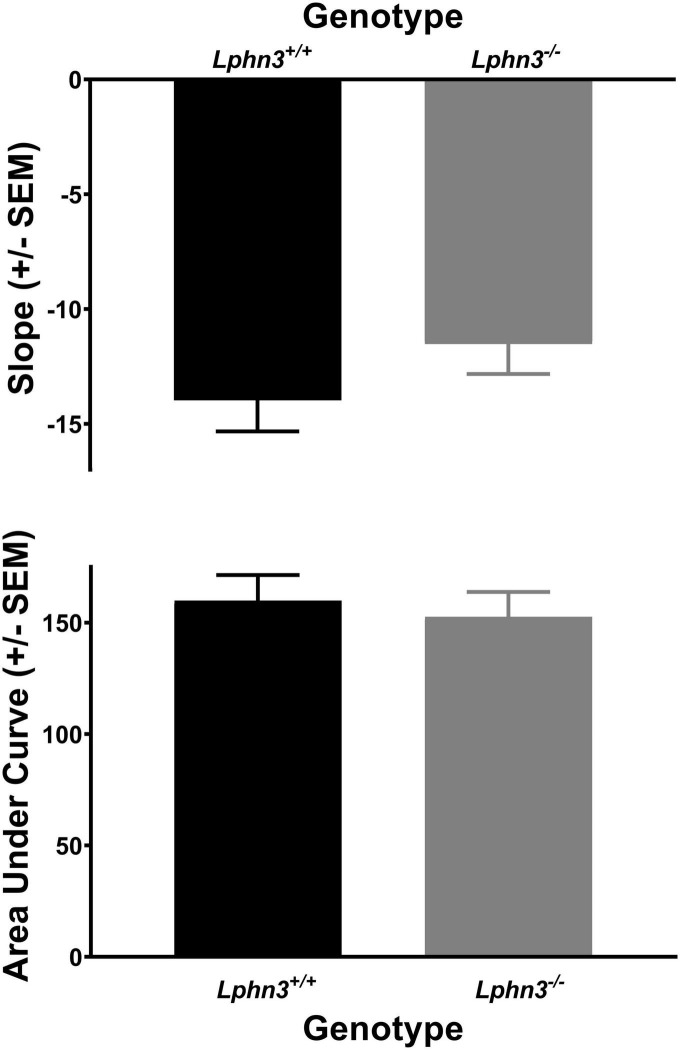
The slope of the discounting curve **(top)** and area under the discounting curve **(bottom)** did not differ between the *Lphn3*^–^*^/^*^–^ (knockout) and *Lphn3*^+^*^/^*^+^ (wildtype) rats with equal trial lengths.

The analysis of the percent choice for the larger reward did not reveal a significant main effect of genotype [*F*(1, 52) = 0.310, *p* = 0.580], nor significant genotype × delay [*F*(2.484, 129.150) = 2.334, *p* = 0.089] or genotype × delay × phase [*F*(2.614, 135.938) = 0.288, *p* = 0.807] interactions. For comparison, Figure 3 shows the results for each genotype across the various delays for both acquisition (top) and maintenance (bottom). As shown in Figure 4, the main effect of genotype was also not significant for the analysis of slope (top) or AOC (bottom) [*F*(1, 52) = 1.693, *p* = 0.199 and *F*(1, 52) = 0.199, *p* = 0.657, respectively].

## Experiment I discussion

We hypothesized that impulsive choice would be greater in the SHR and *Lphn3* KO rats relative to their control strains–WKY and WT rats, respectively. However, the results only partially supported this hypothesis. While the SHRs demonstrated a decrease in the percent choice for the larger reward as well as a more negative slope and decreased area under the curve compared to WKY rats, the *Lphn3* KO and WT rats did not differ on these measures. These results were surprising as both SHRs and *Lphn3* KO rats have been shown to be impulsive when the task assessed impulsive action (Sable et al., 2021).

One possibility is that both the SHRs and *Lphn3* KO rats exhibit impulsive choice, but they present the impairment differently. As previously mentioned, DD deficits have been previously reported in SHRs (Fox et al., 2008; Sjoberg et al., 2021). These results and ours indicate that the SHRs have a substantial problem with delay of gratification. When the delay between response and reward was too long, the greater magnitude of the delayed reinforcer was not enough to entice them to choose that lever. Rather, the delivery of the reinforcer needed to occur soon after lever selection, so they choose the smaller, but immediate reward. As mentioned by Sjoberg et al. DD performance by SHRs strongly supports Dynamic Developmental Behavioral Theory, which argues the salience of a reinforcer decreases as it is separated in time from the response made to achieve it (Sagvolden et al., 2005a; Sjoberg et al., 2021).

Notably, in the version of the task conducted above, a subsequent trial did not begin until the end of the delay period, regardless of which lever was selected. In other words, because rats were required to “wait” for the next trial to start even if they picked the immediate lever, it is possible the *Lphn3* KO rats may have opted to pick the larger magnitude reward. We decided to investigate this possibility in Experiment II.

## Experiment II

Research that has examined whether consistency in trial length affects choice of the lever associated with the larger delayed reward has provided mixed results. For SHRs, the length of the inter-trial interval (ITI) does not appear to influence choice behavior within trials (Sjoberg et al., 2021). However, among ADHD children, when a subsequent trial begins as soon as the reinforcer from the previous trial is delivered/retrieved, this increase in relative response rate has been shown to shift an even greater percentage of responding to the immediate lever, thereby minimizing the impact of reward magnitude (Marco et al., 2009). This finding has also been shown to occur in research animals, especially when the post-reward delay was cued (Pearson et al., 2010). In Experiment II, we incorporated a 1-s ITI immediately after delivery of either the immediate (1 pellet) or delayed (3 pellet) reinforcer. Because previous research has already shown the length of the ITI does not appear to influence choice behavior in SHRs (Sjoberg et al., 2021), we only tested *Lphn3* KO and WT rats in Experiment II. We predicted that the *Lphn3* KO rats would choose the small, immediate reward more often that the larger, delayed reward relative to the WT rats, thereby demonstrating an increase in impulsive choice associated with an increase in the rate of reinforcement.

## Experiment II method

### Subjects

The subjects consisted of an additional 22 *Lphn3* KO rats (11 male, 11 female) and 25 *Lphn3* WT rats (12 male, 13 female) from the Cincinnati children’s transgenic animal and genome editing core that were generated using CRISPR/Cas9 technology as in Experiment I. They were shipped in three cohorts and housing feeding were identical to that employed in Experiment I.

### Apparatus

Behavioral testing was performed in the same automated, rat operant chambers (Med Associates Inc., St. Albans, VT, USA) used in Experiment I. Likewise, white noise was again presented during testing to minimize disruption from outside sounds and Med-PC V software was used to conduct the testing programs and record data.

### Procedure

#### Autoshaping and fixed ratio training

The autoshaping and fixed ratio training programs were identical to those used in Experiment I.

#### Delay-discounting

The DD task used in Experiment II was the same as that used during Experiment I (delays = 0, 4, 8, 12, or 16 s; 10 trials/delay, 50 trials/session), with the exception that a 1 s ITI occurred after delivery of the food reinforcer but before the next trial began regardless of which lever was pressed. Thus, a tendency to respond on the immediate lever resulted in a shorter session duration. Rats completed 25 sessions.

### Design and analyses

As in Experiment I, the percent choice for the larger, delayed reward for the 25 sessions was averaged across blocks of 5 days to yield five, 5-day testing blocks but only data from the first (i.e., acquisition phase) and last testing block (i.e., maintenance phase) were included. The analysis of the percent choice for the larger reward was a 2 (genotype) × 2 (sex) × 2 (phase) × 5 (delay) mixed ANOVA, while slope and AOC were calculated as was done in Experiment I and analyzed separately *via* 2 (genotype) × 2 (sex) × 2 (phase) mixed ANOVAs.

## Experiment II results

**FIGURE 5:**
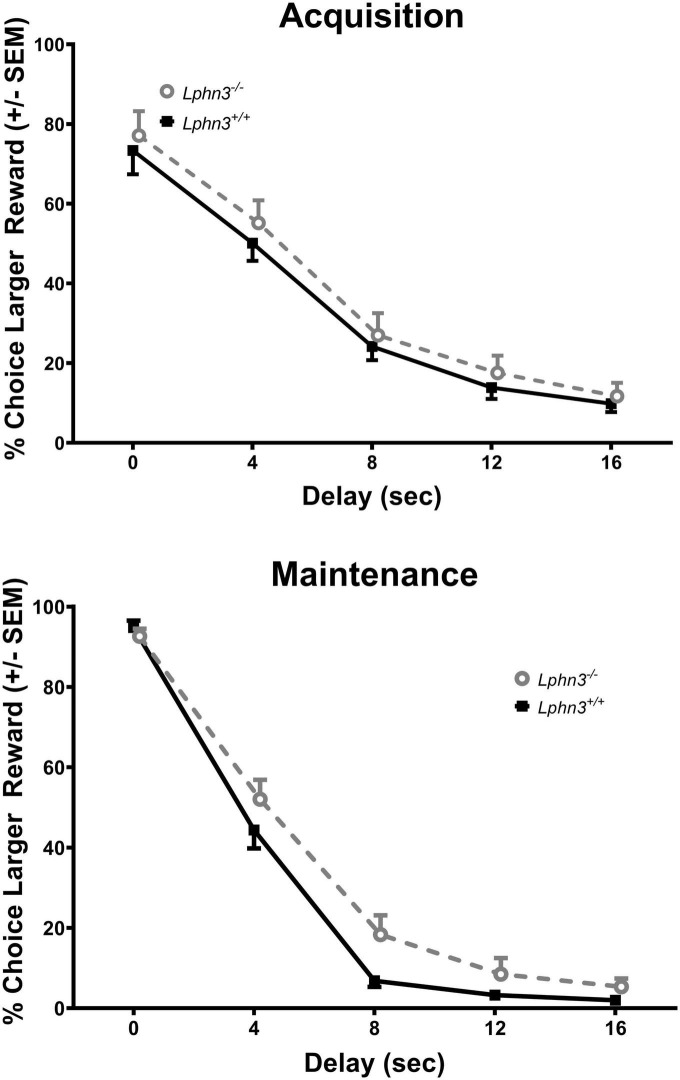
Percent choice for the larger reward during acquisition **(top)** and maintenance responding **(bottom)** for the *Lphn3*^–^*^/^*^–^ (knockout) and *Lphn3*^+^*^/^*^+^ (wildtype) rats with unequal trial lengths. Genotype had no effect of on delay-discounting (DD) performance.

**FIGURE 6:**
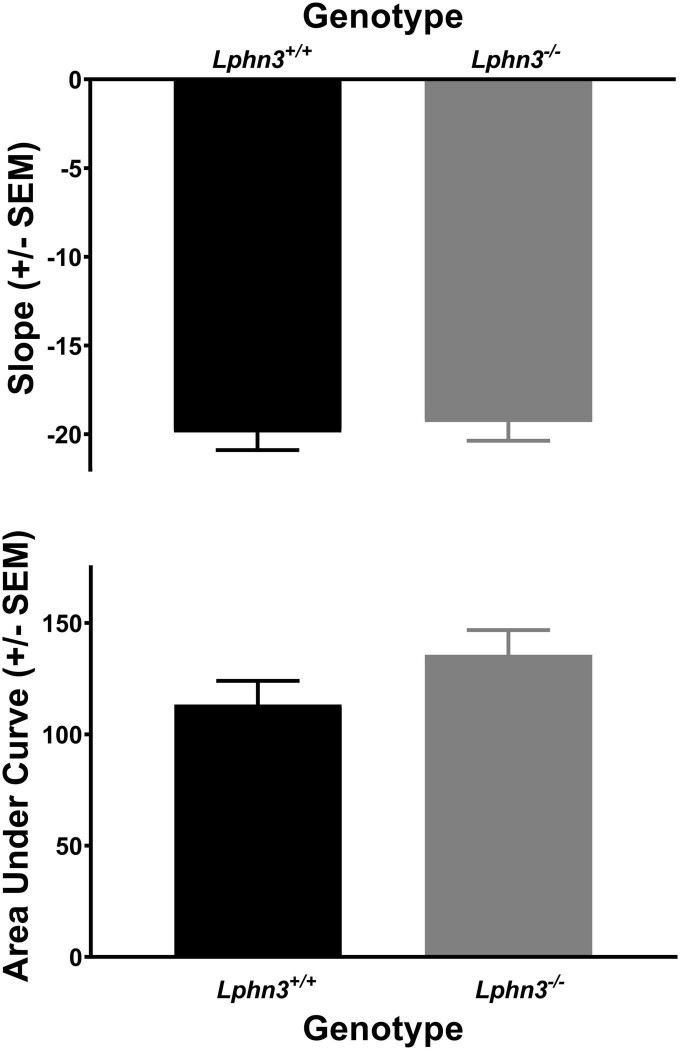
The slope of the discounting curve **(top)** and area under the discounting curve **(bottom)** did not differ between the *Lphn3*^–^*^/^*^–^ (knockout) and *Lphn3*^+^*^/^*^+^ (wildtype) rats when trial lengths were not equal.

The inclusion criterion was the same as for Experiment 1. Data from 2 male *Lphn3* KO rats and 3 *Lphn3* WT rats (2 male, 1 female) were not included as they did not demonstrate 60% choice for the larger reward at the 0 s delay during the final maintenance phase. Thus, 20 *Lphn3* KOs (9 male, 11 female) and 22 *Lphn3* WTs (10 male, 12 female) were included in the final analyses. Greenhouse–Geisser corrections were again used for sphericity violations. Analysis of the percent choice revealed that discounting was evident, as the percent choice for the larger reward decreased overall with increasing delay [*F*(2.034, 77.301) = 337.275, *p* < 0.001]. However, analysis of the percent choice for the larger reward did not reveal a significant main effect of genotype [*F*(1, 38) = 1.462, *p* = 0.234] or sex [*F*(1, 38) = 0.007, *p* = 0.934], nor any significant genotype- or sex-related interactions (see Figure 5). The analyses of the slope and area under the discounting curve also did not reveal significant main effects of genotype [*F*(1, 38) = 0.162, *p* = 0.690 and *F*(1, 38) = 2.070, *p* = 0.158, respectively], nor any other significant genotype- or sex-related differences (see Figure 6).

## Experiment II discussion

The results of Experiment II indicated that removing the post-reward buffer following selection of the lever associated with a smaller, but immediate reward did not differentially affect DD behavior for either genotype. Thus, the rate of reinforcement did not appear to affect impulsive choice in the KO rats. These results are in line with previous research demonstrating that the length of the ITI had little influence on impulsive choice in SHRs (Sjoberg et al., 2021).

## Overall conclusion

Overall, SHRs had increased impulsive choice due to their inability to delay gratification after a response to obtain a reinforcer had been elicited. *Lphn3* KO rats, on the other hand, did not appear to exhibit impulsive choice, even when the option to increase the rate of reinforcer delivery was available. Thus, while both ADHD models exhibit hyperactivity (Russell, 2011; Sagvolden and Johansen, 2012; Regan et al., 2019) and impulsive action deficits (Sable et al., 2021; González-Barriga and Orduña, 2022), impulsive choice appeared to be differentially affected between the models. Notably, our previous research found that while both models exhibited a deficit in impulsive action, the degree of impairment was much more profound in the SHRs than in the *Lphn3* KO rats (Sable et al., 2021). Thus, the overall degree of impulsivity appears to be much more substantial in the SHRs.

Dopamine regulation within the PFC is critically involved in impulsive behavior (Logue and Gould, 2014), and medications targeting the dopamine system are routinely prescribed to ADHD patients in an attempt to reduce impulsive behavior (Arnsten and Pliszka, 2011; Sharma and Couture, 2014). However, as previously mentioned impulsivity is a multi-faceted construct and behavioral deficits on tasks of impulsive choice do not always coincide with deficits on tasks of impulsive action (or vice-versa) in rats or in humans (Solanto et al., 2001; Broos et al., 2012; van den Bos et al., 2014).

Notably, there appears to be some degree of regional specificity that mediates impulsive action versus impulsive choice. While this is not yet entirely understood, in human subjects, gray matter volume in the right frontal pole (RFP) and left middle frontal gyrus (LMFG) were predictive of DD performance, while gray matter volume in the right inferior frontal gyrus (RIFG), supplementary motor area (SMA), and anterior cingulate cortex (ACC) predicted performance on an impulsive action task (Wang et al., 2016). Preclinical research also suggests the RIFG mediates impulsive action along with the ventrolateral prefrontal cortex (VLPFC), while the dorsal lateral prefrontal cortex (DLPFC) and is heavily involved in mediating impulsive choice (see Kim and Lee, 2011; Bari and Robbins, 2013 for reviews).

Our observed differences among the ADHD animal models has the potential to promote a better understanding of the underlying mechanism(s) responsible for the differential behavioral effects observed. The discrepant results between the SHRs and *Lphn3* KO rats presented above suggest more widespread disruption of frontal cortical regions involved in both impulsive action and impulsive choice in SHRs, with disruption limited only to those regions involved in impulsive action in the *Lphn3* KO rats. Ongoing research in our lab is currently investigating this possibility. These findings will be very important as they will have the potential to inform medication development, leading to a more targeted approaches to curb the facets of impulsivity that an ADHD individual may present (i.e., impulsive actions and/or impulsive choice), while sparing those that are not affected. This would undoubtedly reduce side effects and increase compliance.

## Data availability statement

The raw data supporting the conclusions of this article will be made available by the authors, without undue reservation.

## Ethics statement

This animal study was reviewed and approved by the University of Memphis, Institutional Animal Care and Use Committee–protocol #0875.

## Author contributions

MC: data collection, analysis, and manuscript preparation. AB, OH, and HN: experimental design, data collection, and manuscript preparation. SR: experimental design, data collection, and analysis. MW, CV, and HS: research idea, experimental design, data collection, analysis, and manuscript preparation. All authors contributed to the article and approved the submitted version.
