# Supplementary material for: Impulsive choice in two different rat models of ADHD—Spontaneously hypertensive and Lphn3 knockout rats
Source: Front Neurosci. 2023 Jan 26;17:1094218. doi: 10.3389/fnins.2023.1094218 (PMC9909198; doi:10.3389/fnins.2023.1094218)
Supplement: Supplementary file 1 [file Table_1.DOCX]

**Supplemental Table 1. Body Weight Data at the Start of Operant Testing**

______________________________________________________________________________

Mean (g) SEM

______________________________________________________________________________

EXPERIMENT I

SHR

Male (n=11) 202.89 ±6.89

Female (n=9) 156.98 ±6.69

WKY

Male (n=9) 194.44 ±8.47

Female (n=12) 162.90 ±1.78

*Lphn3* KO

Male (n=14) 330.30 ±20.36

Female (n=15) 214.16 ±6.04

*Lphn3* WT

Male (n=12) 391.20 ±24.24

Female (n=15) 233.97 ±9.05

______________________________________________________________________________

EXPERIMENT II

*Lphn3* KO

Male (n=9) 353.80 ±30.44

Female (n=11) 220.27 ±6.65

*Lphn3* WT

Male (n=12) 427.02 ±19.73

Female (n=15) 227.73 ±12.26

______________________________________________________________________________

*Note.* Means (± SEM)
